# [Expression of Concern] lncRNA FLVCR1-AS1 drives colorectal cancer progression via modulation of the miR-381/RAP2A axis

**DOI:** 10.3892/mmr.2025.13774

**Published:** 2025-12-10

**Authors:** Yi Han, Xiaoyan Wang, Enqiang Mao, Boyong Shen, Liang Huang

Mol Med Rep 23: 139, 2021; DOI: 10.3892/mmr.2020.11778

Following the publication of this paper, it was drawn to the Editor's attention by a concerned reader that, regarding the TUNEL assay experiments shown in Fig. 2E on p. 5, the Merge panels for the shFLVCR1-AS1 experiments shown for the Caco-2 and SW480 cell lines appeared to have been inserted into this figure the wrong way around. The authors were contacted by the Editorial Office to offer an explanation for this apparent anomaly in the presentation of the data in this paper; however, up to this time, no response from them has been forthcoming. Owing to the fact that the Editorial Office has been made aware of potential issues surrounding the scientific integrity of this paper, we are issuing an Expression of Concern to notify readers of this potential problem while the Editorial Office continues to investigate this matter further.

